# Substance abuse and psychiatric co-morbidity as predictors of premature mortality in Swedish drug abusers a prospective longitudinal study 1970 - 2006

**DOI:** 10.1186/1471-244X-11-122

**Published:** 2011-07-30

**Authors:** Anna Nyhlén, Mats Fridell, Martin Bäckström, Morten Hesse, Peter Krantz

**Affiliations:** 1 Dept of Psychiatry Lund University Hospital SE - 221 85 Lund, Sweden; 2 Lund University, Dept of Psychology & Vaxjo University, School of Education, Psychology and Sport Science, SE - 35195, Växjö, Sweden; 3 Lund University, Dept of Psychology B 213, SE - 221 00 Lund, Sweden; 4 University of Aarhus, Centre for Alcohol and Drug Research Artillerivej 90, 2300 Copenhagen S, Denmark; 5 Dept of Forensic Medicine Lund University Hospital, S - 221 85 Lund, Sweden

**Keywords:** drug related death, risk factor, gender, competing risks Cox regression, cohort study, Predictors

## Abstract

**Background:**

Few longitudinal cohort studies have focused on the impact of substances abused and psychiatric disorders on premature mortality. The aim of the present study was to identify predictors of increased risk of drug related death and non drug related death in substance abusers of opiates, stimulants, cannabis, sedatives/hypnotics, hallucinogens and alcohol over several decades.

**Methods:**

Follow-up study of a consecutive cohort of 561 substance abusers, admitted to a detoxification unit January 1970 to February 1978 in southern Sweden, and followed up in 2006. Demographic and clinical data, substance diagnoses and three groups of psychiatric diagnoses were identified at first admission. Causes of death were coded according to ICD-10 and classified as drug related deaths or non drug related deaths. To identify the incidence of some probable risk factors of drug related premature death, the data were subjected to a competing risks Cox regression analysis.

**Results:**

Of 561 patients in the cohort, 11 individuals had either emigrated or could not be located, and 204/561 patients (36.4%) were deceased by 2006. The cumulative risk of drug related death increased more in the first 15 years and leveled out later on when non drug related causes of death had a similar incidence. In the final model, male gender, regular use of opiates or barbiturates at first admission, and neurosis were associated with an increased risk of drug related premature death, while cannabis use and psychosis were associated with a decreased risk. Neurosis, mainly depression and/or anxiety disorders, predicted drug related premature death while chronic psychosis and personality disorders did not. Chronic alcohol addiction was associated with increased risk of non drug related death.

**Conclusions:**

The cohort of drug abusers had an increased risk of premature death to the age of 69. Drug related premature death was predicted by male gender, the use of opiates or barbiturates and depression and anxiety disorders at first admission. The predicted cumulative incidence of drug related death was significantly higher in opiate and barbiturate abusers over the observed period of 37 years, while stimulant abuse did not have any impact. Alcohol contributed to non drug related death.

## Background

Drug abusers have an increased risk of premature mortality which is influenced by a number of factors, including types of substances used, patterns of administration, risk behavior, contracted infectious diseases, gender, age and life style.

During the period when the studied cohort entered treatment amphetamine was the single most common substance of abuse in Sweden nationally as well as in the local area, where 35-45% of the drug abusers had amphetamine as the most prevalent substance of abuse [1,2]. However, in the 1970s substance use patterns in Sweden changed from a predominance of amphetamine abuse to include the abuse of opiates as well as other substances [2]. Most studies report higher mortality for opiate users than for other substance users [3-7], but a few studies have found lower mortality rates among the former [8]. The standardized mortality ratio [SMR] in more recent studies is still higher in groups that do not receive opiate agonist treatment such as methadone, buprenorphine or other opioids [7,9-12].

Overdose is a major cause of death among opiate-dependent patients. About 0.7% of opiate dependent users die every year from an overdose [13]. Several studies of clinical samples have reported that 35-40% of all deaths in opiate users are overdoses [3,14-18]. Unlike opiates, cocaine and amphetamine are seldom direct causes of death [13]. Still, stimulant use has been associated with increased mortality, either due to lifestyle factors indirectly associated with stimulant use, such as violent behavior or violent deaths, or with diseases acquired through intravenous administration [19]. A recent epidemiological study of a cohort of 100,000 substance users of amphetamine/methamphetamine and ecstasy in England and Wales 1997-2007 identified an increasing number of amphetamine deaths from 30 to 70 over this period of ten years [20].

Cannabis, on the other hand, is the most common substance worldwide, used by as many as 166 million people per year, but few studies have to our knowledge evaluated mortality associated with cannabis [21]. We know of no studies that have examined the particular long-term risks of death associated with cannabis use in clinical samples of substance dependent patients.

### Influence of psychiatric disorders

The impact of psychiatric disorders on premature mortality in substance abusers has only recently been recognized as an important issue. A few prospective studies have suggested that some psychiatric disorders may contribute to premature death in drug abusers (15). In a Swedish cohort study of drug abusers, low levels of psycho-social functioning measured by Global Assessment of Functioning (GAF) and a high level of psychiatric symptoms assessed by Symptom Checklist 90 (GSI) at the 5-year follow-up, predicted mortality at the 15-year follow-up, whereas abstinence did not [22]. The most prevalent personality disorder in drug abusers; anti-social personality disorder, was not associated with a higher level of premature death [19,22].

### Aims of the study

To identify predictors of increased risk of drug related death and non drug related death within a cohort based on broadly defined psychiatric groups and substance use of opiates, stimulants, cannabis, sedatives/hypnotics, hallucinogens and alcohol over several decades.

## Methods

### Setting and subjects

The setting was an inpatient detoxification and short-term rehabilitation unit. The ward was a typical low threshold unit of the period, accepting all drug abusing patients seeking treatment but only to a minor extent patients with alcohol dependence. The catchment area for the unit was the entire county of Scania in southern Sweden with a population of 977,783 people in 1970, and of 1,126,606 in 2000. Southern Sweden is an urban area which in the early 1970s had a low rate of unemployment (5%) which increased to 11% during the observation period.

A national case-finding study estimated that the number of heavy drug abusers in the southern region having a daily intake of illegal drugs was around 1500 - 2000 persons in 1978, of which 1500 (75%) were injection drug users [23].

All patients admitted to the detoxification unit January 1970 to February 1978 were included in the cohort. The admission was completed only after the patient had had a formal somatic and psychiatric screening and had completed the intake procedures with laboratory specimens. The cohort was followed up through the Swedish Central Person Register, death certificates and autopsy reports were obtained for all subjects who died before 2007, and causes of death were analyzed. For this study all causes of death were coded according to ICD-10, based on autopsy reports or, in a few cases, death certificates in addition to hospital records. The study was approved by the Ethical Committee of Lund University (LU 22/1983 and Dnr 587/2005).

### Assessment at first admission and at follow up

Substance type and other drugs including alcohol were identified with interviews and validated by mandatory urine samples. Demographic data, types of substances used and psychiatric diagnoses were collected in a standardized manner. Standardized clinical interviews (SWEDATE) and hospital records contained background data and mandatory information on length and intensity of substance abuse by mode of administration [24]. At intake all patients received an identification number and gave verbal consent for participating in the study upon completing the admission routines. All patients were evaluated by the senior consultant of psychiatry at the detoxification ward. A clinical psychologist provided additional psychological assessment. Patients with psychosis were evaluated both at the detoxification unit and independently by an external consultant in psychiatry at a special psychosis unit in the same hospital. A formal diagnosis of psychosis demanded two or more treatment admissions or a longer observation period in the unit before a final diagnosis was issued.

The ICD-8 diagnoses were filed at discharge: The psychiatric disorders are in this presentation categorized into three broad groups represented as dummy variables: psychosis, neurosis and personality disorder. The neurosis category included depression (minor and major without psychotic symptoms), and anxiety disorders and a few cases of phobias. The personality disorders were anti-social, hysterical and infantile. For the present analysis drug use was re-coded into dichotomous variables, with 0 representing no use and 1 representing regular use for each drug on a daily basis, or a minimum of three days a week, for at least 12 months.

### Coding and identifying causes of death

In the follow-up study the patients' national identification numbers were linked to the Swedish Central Person Register and the Causes of Death Register. The coverage of deaths in the Swedish Central Bureau of Statistics (SCB) registers is close to 100%. SCB codes causes of death are based upon death certificates, which are issued but not coded by physicians and/or coroners. All causes of death in the cohort were coded (ICD-10) by a senior consultant physician (A.N.) and an associate professor of forensic medicine (P.K.). The causes of death diagnoses issued by the coroner were never changed by the researchers. The diagnoses from the forensic autopsy protocols, including toxicology tests, as well as the death certificates were coded according to ICD-10. ICD codes permit classification of death causes according to the rules specified in the International Statistical Classification of Diseases and Related Health Problems, published by WHO [25]. ICD-10 provides improved coding possibilities for many drugs compared to previous versions of ICD. The first one hundred causes of death were coded independently (by A.N and P.K). For this study, deaths were classified as either drug related or non drug related as defined below. The reliability was good (**κ **= .98). Only 12 out of 204 ratings differed. The final coding used in the analysis was always based on a mutual agreement between the two raters.

#### 1. Drug related death

Our definition of drug related death used is the one adopted by Bargagli [26] and Degenhardt [13]. Drug related death both refers to those cases where the underlying cause of death is directly associated with illicit substance use, sometimes in combination with licit drugs according to death certificates and/or autopsy protocols, and those cases when substance use disorder was listed as a contributing cause of death. Preset rules (a manual of coding) determined if death was drug related. The decision was based on the total amount of data present: hospital records, police reports and coroners' evaluation including toxicology reports, which always took precedence over other documents.

#### 2. Non drug-related death

Non drug related death was classified as such if death was caused by somatic diseases or by accidents, suicide or other violent deaths without illicit or/and licit drugs or alcohol being involved in the death.

### Statistical analysis

The data were subjected to a competing risks Cox regression to analyze the incidence of drug related premature deaths and non drug related deaths with important covariates. Competing risks procedures make it possible to estimate the likelihood of an incidence when other incidences take place that alter the probability of the event of interest. The significance of the covariates is reported, as well as their coefficients (B) and the predicted change in the hazard for a unit increase of the predictor, Exp (B) = Odds Ratios (OR). The competing risks program, developed by Robert Gray for the R statistical package, was used to estimate the coefficients. The text of the program published by Pintilie [27] guided our analysis.

The hypotheses in this study stated that a) different types of diagnostic classes: psychosis, neurosis and personality disorder, as well as b) patterns of substance use at first admission predicted premature death many years later. Two patterns of causes of death were studied: drug related death versus non drug related death. The cumulative incidence of premature death was modeled with these predictors under a competing risk situation. Models for drug related death were compared with models of non drug related causes of death. Next, the subject's gender and age served as covariates in the models. To test the hypothesis, a hierarchical procedure was used starting with age and gender, followed by the three psychiatric diagnostic classes and finally by the substance pattern at first admission.

The predictor variables were selected a priori, and time to death was calculated from the first admission. Since the ethnic diversity of Sweden today is low, (by 2006, 13% were born in another country, most of them in Finland [28] and was even lower in the 1970s, adjusting for race or ethnicity was not considered necessary.

## Results

### Characteristics of the cohort

All patients admitted to the detoxification unit between January 1970 and February 1978 were included (n = 561). The cohort was characterized by a low degree of selection, thus resembling drug patterns prevalent among persons with heavy drug use during the period. Twenty patients who did not complete the intake examination, did not have a correct identification number, nor gave verbal consent were excluded from the cohort. Of the patients included, 31% discontinued the detoxification treatment prematurely, 20% within the first week. However, these individuals went through the same admission procedure as the rest of the cohort, and their data were included in the analyses. There was no association between dropout rate and dominant substance abuse at the first admission. At follow-up 11 of the 561 individuals were not included, due to clerical errors, emigration or by failing to locate them by 2006. The mean observation time was 27.1 years with a range of one to 37 years.

The sample was predominately male, 70%, and almost 90% were young (m = 24.3 years) at first admission to detoxification (Table 1). Regular intravenous illegal substance use was reported in 79% of the patients, and of these were 97% opiate users and 91% amphetamine users. The patients' age at first use of drugs was 15.5 years (MD = 15.0, SD = 3.3) for men and 16.2 (MD = 15.0, SD = 4.7) for women.

**Table 1:** Characteristics of the patients in the cohort at first admission 1970-1978 (n = 561)

	Deceased in 2006	Alive in 2006	Total
**Demographics**	n = 204	n = 357	n = 561
Age at first detoxification	m = 25.9 SD/range 8.8/13-68	m = 23.2 SD/range 5.9/13-50	M = 24,3 (SD = 7.2)
	%	%	%
male gender	76	63	68
**Drugs**			
opiates	82/40%	96/27%	191/34%
stimulants	75/37%	171/48%	236/42%
cannabis	96/47%	218/61%	286/51%
barbiturates	37/18%	32/9%	84/15%
hallucinogenic drugs	18/9%	21/6%	45/8%
alcohol	4/2%	25/7%	28/5%
Poly-drug abuse^1^	120/59%	207/58%	331/59%
**Somatic conditions**			
Any somatic disease	122/60%	182/51%	309/55%
Hepatitis (A, B, non A - non B)^2^	104/51%	164/46%	269/48%
**Psychiatric conditions**			
Psychosis	18/9%	57/16%	79/14.4%
Neurosis	29/14%	54/15%	84/14.8%
Personality disorder	39/19%	75/21%	112/20.1%

^1 ^poly drug use; abuse of at least 2 substances at the same time

^2 ^it was not possible to diagnose hepatitis C before 1991

The drugs most regularly used at first admission, according to urine testing, were opiates (34%), stimulants (42%), cannabis (51%) and barbiturates (15%). About 3% of the patients had chronic alcoholism. Abusing two or more substances regularly (poly drug use) was reported for 59% of the cohort. All substances were proportionally more common among males than among females, but only opiates (p < .02) and cannabis (p < .009) were significant. The deceased individuals were more likely to have used opiates (*χ***^2 ^**= 10,8, p < .002), barbiturates (*χ***^2 ^**= 6,71, p < .01), and alcohol (*χ***^2 ^**= 4,52, p < .03) at first admission compared to those alive in 2006. Patients who were alive at follow-up used amphetamine (*χ***^2 ^**= 4,52, p < .03) and cannabis (*χ***^2 ^**= 13,01, p < .001) to a greater extent.

In a comparison of the cohort characteristics with the case-finding study [23], the age in 1978:was 26 vs 27 years and the dominant pattern of substance abuse: opiates 37% vs 28%, and amphetamine 31% vs 32%. The incidence of injection use was 79% v s 75%. In other words, the profile was reasonably similar. The major difference was that substance abusers admitted to the treatment unit had a higher proportion of persons using cannabis regularly, with 23% vs 8% and more women in the cohort (31%) compared to the case-finding study (23%).

The reliability of the original diagnoses (ICD-8) in the hospital records was good when rated by two independent psychiatrist (**κ **= .97). The most frequent diagnoses in the group of psychoses (14.4%) were schizophrenia (4.5%) and substance-induced (toxic) psychosis (6.5%). The group of patients diagnosed with neurosis (14.8%) according to ICD-8 mainly included patients with neurotic depression (12%, ICD-8 code; 300.40) and/or anxiety (8%, ICD-8 code; 300.00) and a few patients with diagnoses of hysteria, phobias, or obsessive-compulsive neurosis. Affective disorders (ICD-8 codes: 296.00, 298.00) were filed separately. Diagnoses of personality disorder, mainly anti-social personality disorder, were issued for 20% of the cohort (ICD-8 codes 301.00 - 301.99).

### Mortality rates

By 2006, 204 of the 561 patients in the cohort were deceased (36.4%). The age at the time of death for men was 39.9 years (MD = 39.9, SD = 11.9) and 42.9 (MD = 43.9, SD = 14.6) for women. The age of death for the youngest man was 17.9 years compared to the youngest woman (20.9 years). The average age of substance related death was 35.7 years (MD = 34.9, SD = 10.1) and for non substance related deaths 47.6 years (MD = 48.5, SD = 12.7).

The crude annual mortality was 1.3%. The SMR was 5.94 (95% CI = 5.5-6.8), compared to the local gender- and age matched population computed from data issued by the Swedish Central Bureau of Statistics [28].

### Causes of death

Information of date and place of death was available for 204 dead persons in the cohort. Information of the causes of death was missing in two cases. The coding of the 204 deceased individuals was based on forensic autopsy reports (85%) or, to a minor extent, on autopsy reports from general hospitals (5%). Toxicology reports were available for 87%. In 10% of the cases no autopsy report could be retrieved, and the subjects were classified on the basis of death certificates. In addition, data were also obtained from hospital records and police reports. The causes of death for 4 persons who died outside Europe remained inconclusive. The reliability of drug related versus non drug related causes of death was good when rated by the two independent experts (AN) and (PK) (**κ **= .98). The minor inconsistencies between the ratings were considered non-substantial, as they were related to contributing and not underlying causes of death, and the final coding was based on a mutual agreement.

#### Drug related death

Death was drug related in 120 of 204 deaths (59%). Toxicological analyses were available for all of them. In this group 46 deaths were caused by overdoses of illegal drugs, of which 43 (94%) involved opiates and 3 (6%) stimulants. Twenty-nine of 120 deaths (24.2%) were violent ones like suicide, homicide, and accidents. Of the four patients who died within three month after discharge none died from an overdose. About 40% of substances detected at postmortem examinations were illicit and about 60% were licit. Alcohol was found postmortem in 23%.

#### Non drug related death

Somatic diseases (cardiovascular diseases 42%, infections 36%, and cancer 22%) were the primary cause of death in 59 of 84 non drug related deaths (70%) and the primary cause of 30% of all deaths in the cohort (59/204). Other causes of death included suicide, accidents, and homicides.

### Risk factors

#### Gender and age

**Figure 1:**
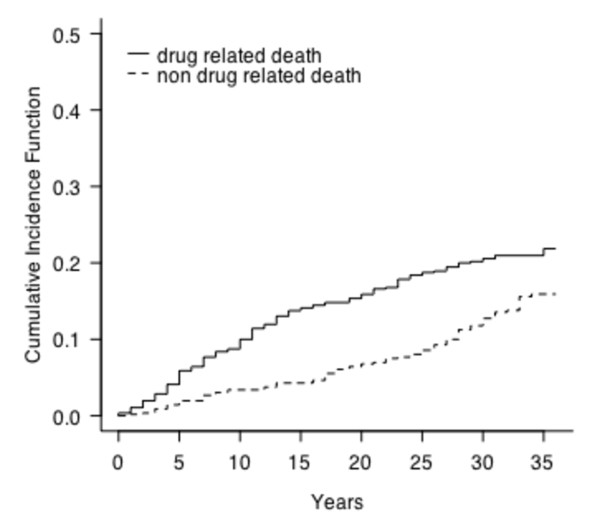
**Patterns of incidence for drug related causes of death versus non drug related causes of death**.

The cumulative incidence of the two competing risks, drug related vs. not drug related, is displayed in Figure 1. The incidence of drug related death increased somewhat more steeply in the beginning of the period, but in the later part of the evaluation period the incidence of non drug related causes of death was similar to that of drug related deaths. As can be discerned in the figure, there was a slight curvilinear association between time and risk of drug related death. The cumulative risk increased more in the first 15 years and leveled out at later points of time. The covariates controlled for in this study were the subjects' gender and age.

Female gender was significantly related to lower risk of drug related death (B = -.50; OR = .61; p = .021), as was the age of the subjects (B = -.03; OR = .97; p > .05). Age was related to non drug related deaths, with an increasing risk with a higher age (B = .09; OR = 1.09; p < .001), with older patients being represented to a higher extent than younger ones.

#### Psychiatric disorders

When psychiatric disorders, here neurosis, psychosis and personality disorder were included together with the subjects' gender and age in the model, only psychosis remained related to drug related death. The incidence of such death was lower in the psychosis group (B = -1.02; OR = .36; p = .009). Personality disorders were not related to drug related causes of death and were consequently dropped in the forthcoming analyses.

#### Drug type/Drug abuse

Next, the drug pattern at first admission was added to the model. Three out of five different drug types were significant. The coefficients from the competing risk analyses are displayed in Table 2. Male gender, higher age and neurosis now became significant predictors of drug related death, while psychosis was only marginally significant (p = .10).

**Table 2:** Competing risk estimates for relevant predictors

Variable	Drug related death B	OR	p	Non drug related death B	OR	P
Gender	-0.733	0.480	**0.001**	-0.298	0.742	0.260

Age	0.033	1.034	**0.042**	0.090	1.094	**0.001**

Psychosis	-0.671	0.511	0.100	0.155	1.168	0.590

Neurosis	0.637	1.891	**0.016**	-0.664	0.515	0.120

Barbiturates	0.330	1.391	**0.002**	-0.189	0.828	0.170

Cannabis	-0.182	0.834	**0.013**	0.020	1.020	0.830

Alcohol	-0.023	0.977	0.900	0.605	1.831	**0.001**

Opiates	0.437	1.548	**0.001**	-0.118	0.889	0.270

Stimulants	0.001	1.001	0.980	-0.125	0.882	0.200

Barbiturates and opiates were related to a higher risk of drug related death, while cannabis was related to a lower risk. The risk of death related to drugs was about 1.55 times higher if opiates was abused at first admission and about 1.39 times higher risk if barbiturates were abused. The risk was 0.87 if cannabis was the main drug abused.

As regards non drug related death, higher age and alcohol were associated with increased risk; the risk of premature death from non drug-related causes was about 1.83 times higher if chronic alcohol problems were present at first admission.

**Figure 2:**
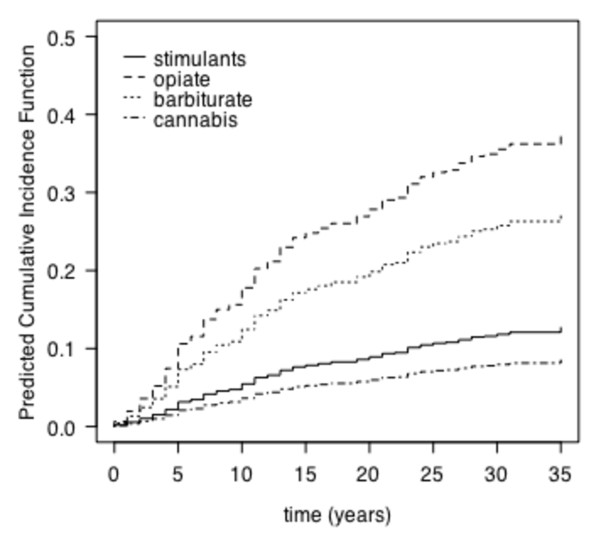
**The predicted cumulative incidences of drug related death for four different drugs tested at first admission and followed over 37 years**.

Figure 2 displays the distribution of risk of drug related death for four drug types over a period of 37 years. The most prevalent groups of drugs are shown. Opiates and barbiturates had a significant impact on drug related death, while cannabis showed a negative association. The use of stimulants had no impact on premature mortality.

## Discussion

The results of the study confirm the long-lasting increased risk for premature death in drug dependent patients. Although the largest risk of drug related death occurred during the first 15 years, the level of such causes of death continued to be high throughout and above the 37-year follow-up period, and premature mortality remained significantly increased up to the age of 69. This stresses the chronic nature of drug abuse and dependence. The age at drug related death in the cohort was 35.7 years, which is close to the mean age of drug induced deaths in some recent European studies [29].

Male gender, opiate and barbiturate use at first admission, as well as neurosis were risk factors for premature drug related death and alcohol use for non-drug related death. Premature mortality was lower in women over time, an observation previously reported in other Scandinavian studies of drug users [4,6,30]. Earlier studies reflecting differences between sexes are not conclusive [3,8,16,19,22]. However, mortality in women in this study was higher in the younger age groups than for men, even though, with regard to the proportion of substances, the age at first use of drugs and age at first admission showed a similar pattern regardless of sex. The relatively lower body weight in women in combination with a propensity to use doses similar to those used by men, might be one explanation for the greater risk of fatal accidental intoxication.

In the European population aged 15 to 49 years, between 10% and 23% of the mortality is attributed to opioid use [26]. Not surprisingly, opiate use predicted drug related death in the studied cohort. In a meta-analysis of mortality the death rates among opiate abusers were about 13 times the norm for their age [31] and 2.4 times higher compared to those of amphetamine users [3]. Fifty-nine percent of the cohort was poly drug users. Mixing several drugs poses a real danger, since non lethal doses of heroin can become lethal in combination with alcohol and sedatives such as benzodiazepines[32]. This study started when barbiturate abuse was common. Barbiturates caused several drug related deaths, mostly in combination with opiates. Both barbiturates and opiates cause respiratory depression which is the major mechanism of opiate death [33]. The use of stimulants had no impact on premature mortality in this cohort. Stimulants do not have the same lethal effects as opiates, but, according to a study of Gossop et al [15], the use of amphetamine in combination with opiates increased the risk of mortality.

Despite the fact that cannabis use in this study did not reflect "recreational use" but a chronic abuse persisting over several years, the association between cannabis use and drug related death was negative. This finding remains in the present cohort even after controlling for the use of other drugs, and support the results of other studies indicating that cannabis is not associated with increased premature mortality [34,35]. It is possible that a passive lifestyle associated with cannabis use in heavy drug abusers exposed these persons to a lesser risk of violent deaths as suicide, homicide and traffic accidents. In support of this suggestion, cannabis abusers from a later cohort from the same hospital showed less risk of committing property and violent crime compared with other types of drug addicts [19]. In contrast, opiate/heroin abuse requires many activities related to pursuing drugs and money by stealing, prostitution or, in some cases, violent offences and, as Hser et al stated in their follow-up study: "heroin addicts also have extensive involvement in criminal activities even into older age" [[36], Pp 308]. However speculative, future research will need to address if cannabis use is also generally associated with lowered risk for overdoses among poly drug abusers.

To our knowledge, no other cohort study of patients with different types of abuse (opiates, amphetamine/stimulants, cannabis, barbiturates, sedative/hypnotics and hallucinogens) has tracked causes of death over almost four decades. Cohort studies of mortality in opiate addicts showed a higher percentage of deceased persons, 58% in a Danish study [9] and 49% in the Californian study by Hser et al [36] compared to the findings of 36% deceased in the present cohort, which included opiates as well as other drugs. Despite variations in time to follow-up, we conclude that the drug use pattern has the strongest impact on drug related deaths.

Half the cohort was diagnosed with psychiatric disorders at first admission. The prevalence of co-morbidity in substance abusers has been reported to increase over the last two decades or longer [37]. The rate of psychoses was however, similar between the present cohort and a later cohort of patients treated from March 1978 to June 1995, while depressions, anxiety and personality disorders became more prevalent [38]. In our cohorts of drug abusers the increase of co-morbidity reflected the more systematic application of diagnostics rather than a general increase in prevalence rates [38].

Two patterns remained in the analysis; neurosis predicted drug related premature death and chronic psychoses did not. The explanation is that only a few patients with chronic psychoses in this study used opiates or amphetamine intravenously. Still, the prevalence of psychotic disorder in this cohort was much higher than in the Lundby population study [39] conducted in the same region. The prevalence of psychoses was at that time 4.2% in the local suburban general population compared to 14.4% in this cohort. The neurosis group included mainly patients with depressive and anxiety symptoms, constituting 15% of the cohort compared to a prevalence of neurosis of 0.4% in the general population [40]. This group of patients could be expected to use more alcohol and sedatives/hypnotics, prescribed or not, for alleviating psychological suffering as a kind of "self-medication", which in combination with opiates increases the risk of premature death. High levels of anxiety have been shown to increase the risk of premature mortality, and regular use of benzodiazepines predicted overdoses in a prospective study of substance abusers in the UK [15]. It is possible that intoxication among suicides may have contributed to the association between neurosis and drug-related premature death. However, given the sample size, having more than two risk outcomes for the competing risks model was not feasible. Future research should investigate this question using larger cohorts.

Some researchers found no association between mortality and psychiatric conditions [9], while others suggest that psychopathology causes increased premature mortality [3,15,22]. Instead of discussing the general impact of the co-morbidity of psychiatric disorders on mortality in drug dependent persons, the case might be that various psychiatric disorders have a differential influence on causes of premature death.

Somatic diseases constituted 70% of the non drug related deaths, and violent death the remaining 30% [41]. In this study alcohol use predicted non drug related deaths. Alcohol dependence is known to contribute to a wide range of somatic diseases, such as liver failure, cancer, coronary diseases, stroke and diabetes. A J-shaped relationship between alcohol and total mortality was confirmed in both men and women in a meta-analysis from 2006 [42]. While moderate consumption of alcohol was inversely associated with total mortality, higher consumption was associated with increased mortality. Illicit drugs contributed to death for those who died from liver failure associated with viral hepatitis and/or chronic alcoholism and for those who died from HIV or HIV-induced opportunistic infections and cancers (AIDS).

Among the strengths of this study are the long observation period and the fact that the cohort was reasonably representative for drug abuse patterns in the southern region of Sweden at the time. According to data from the national case finding study from the end of the 1970s, the cohort was reasonably similar in drug use, age and incidence of intravenous abuse to the population of substance abusers at the time [23]. The slight overrepresentation of women in the clinical cohort was typical for a more pronounced treatment-seeking behavior in women substance abusers compared to substance abusing men [2,6]. In the substance abusing population in Sweden at the time some 25% were women, while in clinical settings women constituted 33% [24]. This was the case also in this cohort.

Causes of death were coded according to ICD-10 classification by a senior consultant physician and an associate professor of forensic medicine, a procedure which eliminated inconsistencies in recording drug related deaths, which are often found when data from national cause of death registers are used as only source. This procedure increased the rate of drug related death by 35% compared to register data only.

There are however some limitations. The first is that the cohort design by necessity provides a more limited number of subjects for analysis, thus restricting its statistical power more than is the case in large epidemiological samples. Secondly, we have not been able to include important aspects of the patients life-situation. Premature death may be predicted by life events like traumas, separation and loss of close friends and relatives, data known to be associated with suicide. Such data were however seldom registered in the patient records in a systematic fashion and have not been included in the analysis.

Thirdly, patients' behavior during treatment as well as their discharge status may be potential indicators of long term risk of premature death. Dropout from treatment is known to increase the risk of death by overdose in opiate abusers [15]. In this study however no overdose was diagnosed in the few patients who died within three months after premature termination of treatment and no association was found between dropout and dominant substance of abuse. Based on the available data, we cannot determine if discharge status at first admission is a predictor of premature death many years later.

Finally, the categorization of co-morbid psychiatric disorders into three broad groups is another limitation. The psychiatric nomenclature used at the time when the patients entered the cohort (ICD-8) might be considered somewhat dated by today's standards. Neurosis, for example, is today replaced by more refined and specific diagnoses of depression and anxiety disorders. Personality disorders had lower prevalence in the cohort than is the case in more recent clinical materials of substance dependent persons [19,22,38]. It is likely that the low prevalence reflected the critical stance of the 1970s drug addiction treatment towards personality assessment in general and psychiatric diagnostics in particular, as articulated by, for example, Thomas Szasz [43].

## Conclusions

About two thirds of all deaths in this cohort of substance dependent persons were drug related. Male gender, abuse of opiates and barbiturates as well as a diagnosis of neurosis, mainly neurotic depression and anxiety at first admission, predicted premature drug related mortality while chronic psychoses and personality disorders did not. The risk of drug related death was about 1.6 times higher if opiates were abused at first admission and about 1.4 times higher risk if barbiturates were abused. The predicted cumulative incidence of drug related death was significantly higher in opiate and barbiturate abusers over the observed time period of 37 years, while stimulant abuse did not have any impact. Alcohol contributed to non drug related death.

## Competing interests

Conflict of interest declaration: The authors declare that they have no financial or other conflicts of interests in relation to this manuscript. The funders had no say with regard to the analyses, interpretation, or decision to submit the manuscript for publication.

## Authors' contributions

MF collected the data and designed the study. Data analyses were carried out by AN, MH and MF. PK provided and coded the autopsy protocols/death certificates together with AN. MB designed and carried out the statistical analyses; AN, MF and MH co-wrote the paper. All authors approved the final manuscript.

## Pre-publication history

The pre-publication history for this paper can be accessed here:

http://www.biomedcentral.com/1471-244X/11/122/prepub
